# Your own actions influence how you perceive other people: A misattribution of action appraisals

**DOI:** 10.1016/j.jesp.2007.11.005

**Published:** 2008-07

**Authors:** Steven P. Tipper, Patric Bach

**Affiliations:** Centre for Clinical and Cognitive Neuroscience, University of Wales, Adelaid Brigantia, Penrallt Road, Bangor, Gwynedd LL57 2AS, UK

**Keywords:** Social perception, Trait attribution, Fluency, Embodiment, Cingulate cortex, Action observation

## Abstract

The attribution of personal traits to other persons depends on the actions the observer performs at the same time (Bach & Tipper, 2007). Here, we show that the effect reflects a misattribution of appraisals of the observers’ own actions to the actions of others. We exploited spatial compatibility effects to manipulate how fluently—how fast and how accurately—participants identified two individuals performing sporty or academic actions. The traits attributed to each person in a subsequent rating task depended on the fluency of participants’ responses in a specific manner. An individual more fluently identified while performing the academic action appeared more academic and less sporty. An individual more fluently identified while performing the sporty action appeared sportier. Thus, social perception is—at least partially—embodied. The ease of our own responses can be misattributed to the actions of others, affecting which personal traits are attributed to them.

Humans constantly attribute personal traits to others. One person may appear intellectual, but not interested in sports, whereas another person may appear more interested in sporting pursuits than intellectual challenges. These attribution processes are quick and automatic (Ambady & Rosenthal, 1992; Bodenhausen & Macrae, 1998; McNeill & Burton, 2002) and rely mostly on salient characteristics of the observed individuals: a person may be tall, strong, and fast; he may play for the college football team but is rarely seen in the library.

Recently, however, researchers have begun to argue that social perception was not only based on readily apparent third-person information, but also on a process of ‘simulation’. Accordingly, people covertly imitate the bodily states of others (Gallese, Keysers, & Rizzolatti, 2004; Prinz, 1997; Wilson & Knoblich, 2005). These embodied person representations could then be used to attribute intentions, emotions, and personal traits to the persons observed. This is possible because people have intimate knowledge about the specific internal states that would generate the bodily states in themselves (e.g., Barsalou, Niedenthal, Barbey, & Ruppert, 2003; for a critical evaluation see Jacob & Jeannerod, 2005).

There is ample evidence that covert imitation takes place in social interactions. Humans non-consciously and non-strategically mimic the people they interact with (e.g., Chartrand & Bargh, 1999; Van Baaren, Holland, Kawakami, & van Knippenberg, 2004; for a review, see Niedenthal, Barsalou, Winkielman, Krauth-Gruber, & Ric, 2005). Similarly, it is known that observed actions prime similar actions in the observer, even when task irrelevant (e.g., Bach, Peatfield, & Tipper, 2007; Brass, Bekkering, Wohlschläger, & Prinz, 2000). Analogous results come from neuroimaging techniques. So-called ‘mirror neurons’ have been discovered in the macaque premotor cortex (DiPellegrino, Fadiga, Fogassi, Gallese, & Rizzolatti, 1992; Gallese, Fadiga, Fogassi, & Rizzolatti, 1996) that fire both when the monkey performs a particular action and when it observes the action being performed by a conspecific. These findings provided a neuronal foundation of the mimicry effects because they reveal how viewed actions can be matched directly to the actions an observer can produce (for similar data in humans, see Grèzes, Armony, Rowe, & Passingham, 2003; Iacoboni et al., 1999).

In recent years, however, it has become clear that embodiment effects are not restricted to the representation of the particular motor acts others perform but also involve their attentional, somatosensory, and affective responses. Observers seem to mimic, for instance, other persons’ gaze shifts (Frischen, Bayliss, & Tipper, 2007), the emotional consequences of others’ pain (Morrison, Lloyd, Di Pellegrino, & Roberts, 2004), and even high-level action control processes evoked by the errors others make (Schuch & Tipper, 2007; Van Schie, Mars, Coles, & Bekkering, 2004).

Even though there is now ample evidence for automatic covert mimicry processes, it remains unknown whether these processes can form the basis of person judgments. A critical test would be to investigate whether changes in the bodily states of the observer can give rise to changes in how other people are perceived. There are studies that demonstrate the ‘projection’ (Freud, 1915/1953) of an observer’s traits onto others. For instance, Kawada, Oettingen, Gollwitzer, and Bargh (2004) showed that traits such as competitiveness and belief in malleable intelligence can become transferred from self to others, even when only held implicitly. However, even though these studies demonstrate that traits of an observer can become misattributed to other persons, they say nothing about whether such misattributions can be evoked directly by an observer’s bodily experiences, as would be predicted if judgments of others were based on a covert imitation of their bodily states.

Recently, we have provided evidence for a very specific effect of action on social perception (Bach & Tipper, 2007). We asked participants to identify two actors—‘George’ and ‘John’—by responding with either their finger or their foot. Both actors were shown engaging in the sporty action of kicking a soccer ball and the academic action of typing on a computer keyboard. Thus, depending on whether an actor had to be identified with the finger or the foot, participants’ responses were either similar to the actor’s sporty action and dissimilar to his academic action, or vice versa. We investigated whether this similarity affected how ‘sporty’ and how ‘academic’ the two actors were subsequently perceived to be.

The results of the identification task replicated previous research on imitative behavior as reviewed above, showing that responses were faster and more accurate when they were similar to the observed action. Interestingly, the fluency of participants’ responses also affected how ‘sporty’ and how ‘academic’ the two actors were perceived to be. An actor identified with a finger response was not only identified more quickly and accurately while performing the academic action of typing on a keyboard than the sporty action of kicking the soccer ball, he was subsequently judged to be more academic and less sporty. In contrast, an actor identified by a foot response was identified more fluently when kicking a soccer ball than when typing on a keyboard. He was later perceived to be sportier and less academic.

The finding that our actions affect the personal traits we attribute to other people demonstrates that social judgments rely on representations in our own action system. However, it is unresolved on which level these effects occur. There are two possibilities:

First, effects may only emerge during the representation of specific motor acts (e.g., kicking and typing actions). If self-produced and observed motor acts activate overlapping representations, any representation of an observed action should be enhanced if the observer performs a similar action, and disrupted if she/he performs a different action (e.g., Barsalou et al., 2003). Such effects have been observed before (for a review, see Niedenthal et al., 2005). For instance, people found a message more agreeable if they nodded their heads while receiving it than when they shook their heads (Wells & Petty, 1980), and inducing smiles or frowns affected how funny participants rated cartoons they saw at the same time (Strack, Martin, & Stepper, 1988). In a similar fashion, identifying an individual with a finger response would, on the one hand, interfere with the representation of the (dissimilar) kicking action and make him appear less sporty. On the other hand, the same finger response would enhance the representation of his (similar) typing action so that the individual appears more academic. This motor act hypothesis predicts that the effects on personal trait judgments emerged because there were different amounts of similarity between the sporty and academic actions participants saw and the responses they made at the same time.

Humans do, however, represent actions not only in terms of the specific motor acts, but also evaluate actions in terms of outcomes: whether they can be performed fluently, or whether they are associated with increased effort, pain or errors (for a review, see Botvinick, Cohen, & Carter, 2004). Our personal-trait judgment effects might therefore also reflect that participants misattributed such appraisals of their own responses to the actions of the individuals. Recall that the similarity of the self-produced and observed action was associated with fewer errors and a faster speed of the participants’ identification responses. An actor identified with a foot response might therefore have appeared sportier because he was identified more fluently when he was seen in the sporty situation than when he was seen in the academic situation. Conversely, the other actor might have appeared more academic because he was identified with a finger response more fluently when seen in the academic situation. This view predicts that the similarity of one’s own responses and observed actions is not critical. Rather, it is important that the participants’ responses differ in degree of fluency when actors are seen performing the sporty and academic actions.

To test whether changes in response fluency suffice to induce changes in personal trait judgments we adapted our original paradigm (Bach & Tipper, 2007). We kept the similarity of observed and self-produced actions constant, and induced changes in response fluency by exploiting spatial compatibility effects. In these spatial compatibility effects responses are faster and more accurate when they occur on the same side as the eliciting stimulus (Simon, Craft, & Small, 1971; Simon & Rudell, 1967). If the personal trait judgment effects are due to the ease of participants’ responses being misattributed to the observed actions then they should also occur in the present experiment. If, however, the personal trait effects require overlapping representations of observed and self-produced motor acts, effects on personality judgments should not be obtained.

## Experiment

In the experiment, participants made left and right finger key presses to identify two actors (George and John) that were presented either kicking a soccer ball or typing on a keyboard. To do this, the participants had to orient visual attention to the faces of the actors. Presenting this critical stimulus feature either to the left or right side of the screen allowed us to manipulate its spatial compatibility with the left or right keys used to identify the actors, thereby inducing changes in response fluency (the Simon effect; Simon & Rudell, 1967; Simon et al., 1971), without affecting the similarity between observed actions and the participants’ responses.

**Fig. 1:**
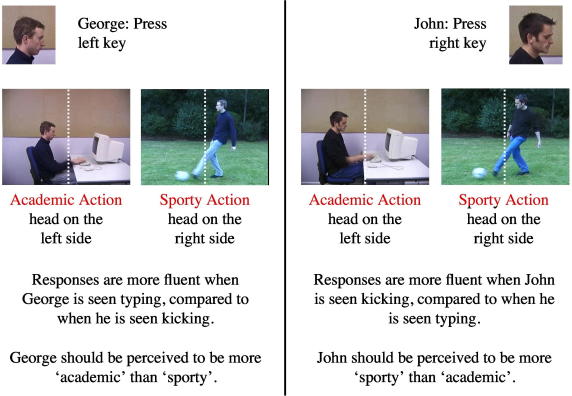
This figure shows the individuals to be identified, typical displays and the basic design. The dotted white vertical line is shown to depict the centre of the display for the reader. It was not present in the stimulus displays.

Assume, for instance, that for a given participant both actors are always presented on the right when performing the sporty action and on the left when performing the academic action (see Fig. 1). This creates a situation in which the response to identify one of the two actors (e.g., the right key press to identify John) will be spatially compatible, and hence faster and more accurate, when he is seen in the sporty context and incompatible, and hence slower and less accurate, when seen in the academic context. Of course the opposite pattern should be observed for the left key-presses to identify George.

The critical question is whether induced differences in response fluency will translate into differences in personal trait judgments. If the effects on personal trait judgments are due to a misattribution of action appraisals, then the actors should take on the traits associated with the situation in which they are most fluently identified. In the above example, an actor should appear more academic when he is identified with a left key because this response is spatially compatible with the location of his face in the academic scenes (‘George’ in Fig. 1). An actor should appear sportier when he is identified with a right key because the right key is spatially compatible with his position in the sporty scenes (‘John’ in Fig. 1).

Note that any changes in personal trait judgments induced in this way cannot be attributed to a similarity between observed actions and self-produced responses. Each actor (George or John) is associated with a finger key press response, as is each action (sporty and academic).^1^ Thus, if effects on personal trait judgments require that self-produced and observed actions converge on the same representations (as assumed by the motor act hypothesis) then no such effects would be expected in the present experiment.

### Method

#### Participants

Thirty-two students (27 females) ranging in age from 18 to 42 years participated in the study. All participants had normal or corrected-to-normal vision. The key assignment of actors (George/John) to response keys (left/right) was counterbalanced across participants, as was the side on which the persons appeared in the scenes (left in the sporty scenes and right in the academic scenes, or vice versa). Thus, for one half of the participants, John was identified with a compatible response when typing and George when kicking, and vice versa for the other half of participants. Participants satisfied all requirements in volunteer screening and gave informed consent approved by the School of Psychology at the University of Wales, Bangor and the North-West Wales Health Trust, and in accordance with the Declaration of Helsinki.

#### Material and apparatus

Participants were seated in a dimly lit room facing a color monitor at a distance of 60 cm. The experiment was controlled by Presentation run on a 3.0 GHz PC running Windows XP. The stimulus set was identical to Experiment 1 of Bach and Tipper (2007). It consisted of eight movies (see Fig. 1 for examples) lasting 1100 ms each and subtending eight degrees visual angle vertically and 11 degrees horizontally. Two of these movies showed John or George kicking a soccer ball, and two movies showed John or George pressing a key on a computer keyboard. In these four movies, the head of each actor appeared on the left side of the frame centre (eccentricities: kicking: 1.1 degrees; typing: 1.9 degrees). For each of these movies a mirror-inverted version was created, in which the actor’s face appeared on the right.

#### Procedure and design

After the computer-driven instructions and a short training phase of 16 trials the experiment began. It lasted for about 15 min and consisted of 320 trials. Each participant saw four of the eight movies, which were presented at equal rates in a randomized order. Movies were selected in the following way. For one participant, the two actors were presented on the right while kicking and on the left while typing. For another participant, the sides of presentation were reversed between typing and kicking actions. This ensured that for each participant, a right key-press to identify actor one was spatially compatible with only one of the two situations in which he appeared (e.g., the sporty situation of kicking a football) and spatially incompatible with the other situation (e.g., the academic situation of typing). Conversely, the left key-press to identify actor two was spatially compatible with the academic situation but not the sporty situation.

Participants initiated each trial by pressing the space bar with their left hand. After 500 ms the movie was presented. Participants identified John or George by pressing either the / or ⧹ keys on the computer keyboard with their left or right index finger. Participants were instructed to identify the individual during the interval in which the movie played (1100 ms). If their identification was correct, the next trial was allowed to start. If participants were too slow or committed an error an error-message was displayed.

After the experiment was finished, a short questionnaire consisting of four questions was presented on the computer screen. Participants were presented with the name and face image of one actor and asked to indicate on a scale from −4 (‘not at all’) to 4 (‘very much”) how sporty they imagined him to be. They also rated the degree to which they thought him academic. They answered the same two questions with regard to the second actor. The order in which actors and traits were rated was counterbalanced across participants.

### Results

#### Vision-action fluency

RTs were entered into a repeated measures ANOVAs with the within-subjects factors Observed Action (sporty/academic) and Person (John/George) and the between-subjects factor Compatibility (whether John is spatially compatible when typing and George when kicking, or vice versa). Trials in which participants were too slow or in which they pressed a wrong button were excluded (4%). The analysis revealed main effects of Person (*F*[1, 30] = 9.3, *p* < .005) and Observed Action (*F*[1, 30] = 24.1, *p* < .0001). John was generally identified faster than George, and the persons were generally identified faster in the academic scenes, in which the faces were larger/clearer. Most importantly, the predicted three-way interaction of Person, Observed Action and Compatibility was highly significant (*F*[1, 30] = 21.3, *p* < .0001). Thus, the RTs to identify the two persons in the two situations depended on which person was identified with a spatially compatible response while typing, and which person was identified with a compatible response while kicking.

**Fig. 2:**
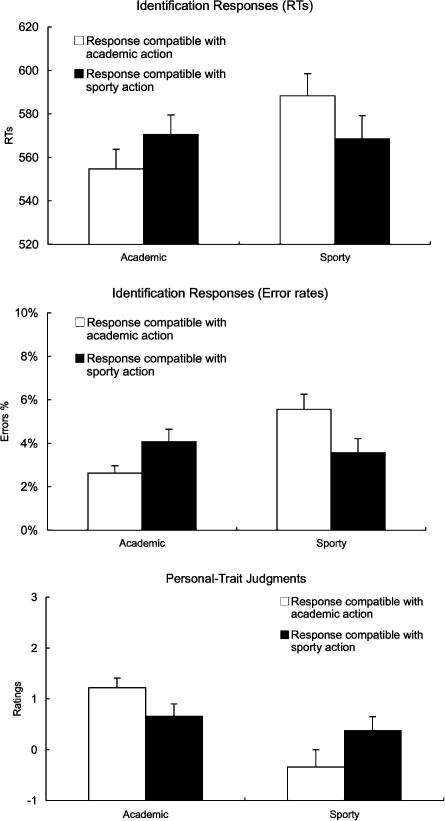
Results. The upper two panels show the spatial compatibility effects for RTs (upper panel) and Error rates (middle panel) in the identification task, collapsed across the two persons (John and George). The lower panel shows the results of the personal trait judgment task. Error bars show the standard error of the means.

We further investigated whether this dependency on compatible responses was present for both the sporty and academic situations. A two-way ANOVA with the within-subjects factors Person (John, George) and the between-subjects factor Compatibility (whether John or George is compatible while typing) computed for the RTs in the academic scenes indeed revealed the critical two-way interaction of Person and Compatibility (*F*[1, 30] = 9.8, *p* = .004), with responses being generally faster for the person identified with a compatible response while typing. The reverse result was obtained for the analogous analysis of the sporty scenes (*F*[1, 30] = 13.0, *p* = .001), with faster responses for the person identified with a compatible response while kicking. See Table 1 for the RT data in all conditions, and Fig. 2, top panel, for the data collapsed across George and John.

Error rates were analyzed with the same ANOVA model. There was a main effect for Observed Action (*F*[1, 30] = 8.4, *p* = .007), that was further qualified by an interaction of Observed Action and Person (*F*[1, 30] = 7.2, *p* = .011). The persons were more easily identified in the academic scenes than the sporty scenes, and this advantage was particularly found for the identification of George. The critical three-way interaction of Observed Action, Person, and Compatibility was again significant (*F*[1, 30] = 18.8, *p* = .0001). Again, the dependency on spatial compatibility was present for both sporty (*F*[1, 30] = 9.1, *p* = .005) and academic scenes (*F*[1, 30] = 6.0, *p* = .021). Thus, in both scenes, the participants made fewer errors when identifying the person for which response side and head location were compatible (see Fig. 2, middle panel, and Table 1).

#### Personal-trait judgments

As in the RTs and Error rates, the rating data were entered into a repeated measures ANOVA with the within-subjects factors Trait (sporty/academic) and Person (John/George) and the between-subjects factor Compatibility (whether John is spatially compatible when typing and George when kicking, or vice versa). The results mirrored the original study (Bach & Tipper, 2007). Main effects of Trait (*F*[1, 30] = 21.0, *p* < .0001) and Person (*F*[1, 30] = 25.6, *p* < .0001) reflected that overall, the persons were judged more academic than sporty, and that John received higher ratings than George. There was also a Person by Trait interaction (*F*[1, 30] = 46.8, *p* < .0001) indicating that the two persons were rated differently on the two traits: John was judged sportier than George (*p* < .0001), but George appeared more academic (*p* < .0005).

Most importantly, as in the RTs and Error rates, there was a three-way interaction of Person, Action, and Compatibility (*F*[1, 30] = 6.8, *p* < .014). Thus, the attribution of personal traits to the two persons depended on which person was identified with a spatially compatible response while typing, and which person was identified with a compatible response while kicking. Two-way ANOVAs showed again that this dependency on compatible responses was present for both academic (*F*[1, 30] = 4.4, *p* = .044) and sporty judgments (*F*[1, 30] = 6.4, *p* = .017). Although George was generally seen to be more academic than John, this difference was reduced when John was identified with a compatible response while typing. Conversely, although John was generally perceived sportier than George, this difference was reduced when George was identified with a compatible response while kicking. Fig. 2, lower panel, shows the rating data collapsed across John and George, and Table 1 shows the data in all conditions.

#### Mediational analysis

As a last step of our analysis, we performed a mediation analysis to investigate whether our spatial compatibility manipulation affected trait judgment directly or by inducing changes in response fluency during person identification. To this end, we derived single measures for (a) the spatial compatibility manipulation, (b) the resulting compatibility effects in the RTs and Error rates, and (c) the compatibility effects in the personal trait judgments. The measure for the spatial compatibility manipulation was derived by setting the value to 1 for the participants for whom George was compatible when typing and John when kicking, and to −1 for the participants with the reverse assignment. The measure for the compatibility effects in RTs, Error rates and trait judgments were calculated by subtracting the mean of the sporty John and academic George responses from the mean of academic John and sporty George responses. The first group of participants should therefore show positive compatibility effects, whereas the second group should show negative effects.

Correlational analyses revealed first that, as shown in the main analysis, spatial compatibility was correlated with trait judgments effects (*r* = .43, *p* = .014) and with the fluency effects in the identification task (RTs, *r* = .64, *p* < .001; Errors, *r* = .62, *p* < .001). Consistent with our mediational hypothesis, the fluency effects in the identification task were in turn positively correlated with subsequent trait judgment effects, though this relationship was only significant for the Error rates (*r* = .45, *p* = .01) but not for the RTs (*r* = .22, *p* = .23).

The critical test for mediation is whether controlling for the effects of the mediator variables (i.e., the fluency effects) significantly reduces the relationship between spatial compatibility and trait judgments (i.e., the Sobel test; Sobel, 1982; for a review see Preacher & Hayes, 2004). The data were therefore entered into a multiple mediator regression analysis (for details, see Preacher & Hayes, in review) that uses the fluency effects in both RTs and Error rates as potential mediator variables, so that the unique effect of each variable could be captured while the other variable was controlled (e.g., Preacher & Hayes, in review; Shrout & Bolger, 2002). This analysis indeed revealed a marginally significant mediation effect for the fluency effect in the Error rates (*z* = 1.9; *p* < .07), but not for the effect in RTs (*z* = −1.4; *p* = .17). Thus, the mediational analysis confirms that our spatial compatibility manipulation affected trait judgments not directly, but at least partially by inducing fluency effects in the Error rates during person identification.

## Discussion

The present study replicated the vision-action personality effect demonstrated by Bach and Tipper (2007). As in the previous study, the actions of the observer influenced which personal traits she attributed to individuals she watched at the same time. As such, social perception appears to be at least partially grounded in the system that we use to perform and to represent the outcomes of our own actions (Bach & Tipper, 2007; Niedenthal et al., 2005).

Our new results also provide insights into the level of action representation at which the effects occurred. They point towards a misattribution of high-level appraisals of one’s own actions to the actions of others. We manipulated the fluency of the participants’ responses by varying the spatial compatibility of the response keys (left/right) and the position of the actor’s face in the scenes (left/right). These variations in response fluency were sufficient to influence personal trait judgments. Actors were judged sportier when they were identified more fluently while performing the sporty action. They were judged more academic when they were more fluently identified while performing the academic action.

The critical role of fluency in affecting personal trait judgments was further confirmed by a mediational analysis. It showed that our spatial compatibility manipulation did not influence subsequent personal trait judgments directly, but specifically because it evoked changes in the fluency of the responses. As in the previous study (Bach & Tipper, 2007), this relationship between fluent responses and subsequent trait judgment effects was specifically found for the Error rates, for which fluency changes were very salient due to error feedback, but not for the more subtle changes in response speed.

These observations are not consistent with our prior assumption (Bach & Tipper, 2007). We had assumed that the judgment effects occurred because observed and self-produced motor acts converged on the same representations in the so-called ‘mirror’ areas of the brain. One person might, for instance, have appeared sportier because the representation of his kicking action was enhanced if the observer performed a (similar) foot action, and disrupted if he performed a (dissimilar) hand action. Although such a process could take place, it cannot explain the present results. Personal trait judgment effects were evoked even though there was no differential amount of similarly between the participants’ left and right responses and the sporty and academic actions. Our new results therefore indicate that the effects occurred on a higher level of action representation, reflecting a misattribution of appraisals of the observer’s own actions to the actions of others. In particular, they indicate that fluency appraisals of the observer’s own actions might become misattributed to the actions of others and affect how they are perceived.

Similar effects of fluency on judgments have been observed before. A robust finding is that stimuli that are more fluently identified also appear more aesthetically pleasing (for a review, see Reber, Schwarz, & Winkielman, 2004). However, our results go beyond these findings in several respects. First, whereas in previous studies fluency was a consequence of the perceptual properties of the viewed stimuli such as contrast or presentation time, it was now manipulated by affecting the overt motor behavior of the participants (i.e., the speed and accuracy of their identification responses). Second, previously observed changes in attitude to objects were typically very general, affecting global attributes such as liking or beauty. In contrast, the present manipulation induced specific changes in attitude and enhanced certain traits of the observed persons but not others (i.e., a person appears more sporty but less academic, or vice versa). And third, to our knowledge, this is the first study that used a motor fluency manipulation to successfully induce changes in the attitude towards other people, as opposed to abstract stimuli or objects.

Our new findings may serve to link current ideas from social psychology to research on clinical populations. A failure to differentiate self from other is increasingly recognized as a hallmark of various clinical syndromes. For instance, autistic individuals exhibit a number of behaviors that suggest a failure to distinguish self and other, such as echolalia and echopraxia, difficulties in theory of mind tasks, and the confusion of the pronouns “I” and “You” (cf. Rogers & Pennington, 1991; Russell & Jarrold, 1999). Similarly, in schizophrenia the inner speech of the sufferers might become misattributed to other, often malevolent, individuals (cf. Frith, Rees, & Friston, 1998). Our study shows that such misattributions are not restricted to clinical populations but also take place in the general population, though of course to a less extreme extent.

The differences between our study and previous reports of fluency affecting stimulus judgments also raise the question about which mechanism drives the effect, and about exactly what becomes misattributed. Prior research on fluency suggests that the effects occur on the level of affective responses (for a review, see Reber et al., 2004). For instance, Monahan, Murphy, and Zajonc (2000) showed that presenting stimuli repeatedly leads to a general enhancement of positive affect that, in turn, can affect the judgment of even unrelated stimuli. Similarly, the fluent identification of one individual (e.g., John) in one of the situations (e.g., sporty) might have evoked positive affective responses, which in turn influenced how the individuals were perceived. It could therefore be that affective responses are at the core of a general mechanism that affects appraisal processes, influencing judgments of one’s own actions, the actions of others, and even of non-animate objects in the environment.

A second possibility is that the personal trait judgment effects reflect the misattribution of the fluency experiences themselves, regardless of affective consequences of the fluent responses. This idea relies on the assumption that people constantly evaluate their own actions and that these evaluations can ‘spill over’ to the actions of others, thereby affecting trait attributions based on these actions. Judgment effects that originate from evaluations of the observer’s own actions are not unknown in social psychology. Higgins has introduced the notion of ‘value from fit’ (e.g., Higgins, 2000). If an action is appropriate to an internal state, this creates a feeling of ‘rightness’ that can transfer to unrelated stimuli and make them appear more valuable. Interestingly, this effect has been shown to be independent of mediating affective factors (Higgins, Idson, Freitas, Spiegel, & Molden, 2003). We propose that the fluency experiences evoked by spatially compatible responses might similarly be transferred to the observed individuals and let *their* actions appear more fluent.

Recent findings from neuroimaging studies are consistent with such a view. Areas in the anterior cingulate cortex (ACC) have been shown to evaluate the outcome of actions with regard to effort, pain, errors, or the presence of response conflict (for a review, see Botvinick et al., 2004). Intriguingly, it has recently become clear that the ACC has mirror properties, that is, it represents these properties for own and others’ actions alike (Morrison et al., 2004; Van Schie et al., 2004; Schuch & Tipper, 2007). This overlap in the neuronal representations of the *evaluation* of one’s own and other’s actions, such as effort, errors or conflict, is different from the *specific* motor processes simulated when observing another’s action, such as whether a hand or foot is used, assumed by the mirror neuron theorists.

## Conclusions

Humans attribute personal traits to others on the basis of action representations that code for both the observer’s own actions and the actions of others. However, the critical overlap between self and other did not exist on the level of the specific motor acts that were performed. Rather, the present results indicate that the *outcomes or appraisals* of one’s own actions were misattributed to the actions of others. These findings are consistent with embodied accounts of social perception that do not restrict mirroring to the level of motor representations, but that assume that all aspects of another person’s state can be represented as if they were one’s own, including high level evaluative and affective responses.

## Figures and Tables

**Table 1:** Results of the experiment in RTs, Error rates, and personal trait ratings for the two persons (George, John) and both scenes (academic, sporty), depending on whether George was always presented in a compatible manner while typing and John while kicking, or vice versa

	George compatible when typing				John compatible when typing			
	John compatible when kicking				George compatible when kicking			
	Sporty		Academic		Sporty		Academic	
	George	John	George	John	George	John	George	John
RTs (ms)	600	**564**	**563**	573	**573**	576	568	**547**
*SD*	45	**42**	**37**	38	**75**	67	63	**62**
Errors (%)	5.9	**2.9**	**1.9**	4.4	**4.2**	5.2	3.8	**3.4**
*SD* (%)	4.4	**2.5**	**1.6**	3.1	**4.5**	3.6	3.5	**2.0**
Ratings	−1.9	**1.3**	**1.7**	−0.1	**−0.5**	1.3	1.4	**0.8**
*SD*	1.0	**1.1**	**0.6**	1.1	**1.4**	1.2	1.3	**1.2**

Bold numbers indicate compatible responses.

## Footnotes

1 Note that the stimuli also contained actions towards the left or right that could, in principle, produce compatibility effects with the side of the response. However, to perform the identification task, participants had to orient their attention towards the faces of the actors. Since compatibility effects strongly depend on where attention is distributed on the display (e.g., Bach et al., 2007), the side of the actors’ head should determine compatibility effects to a stronger extent than the direction of the irrelevant action. Moreover, the direction of the action was always opposite to the side of the individuals’ heads in the displays. Action based compatibility should therefore, if anything, produce the *opposite* pattern of results (in RTs, Error rates, and ratings) as the spatial compatibility between head location and response keys.
